# Enterococcal endocarditis management and relapses

**DOI:** 10.1093/jacamr/dlae033

**Published:** 2024-03-06

**Authors:** Nina Garofoli, Véronique Joly, Diane Le Pluart, Claire Amaris Hobson, Anne-Lise Beaumont, Sylvie Lariven, Nathalie Grall, Marylou Para, Yazdan Yazdanpanah, François-Xavier Lescure, Nathan Peiffer-Smadja, Laurène Deconinck, Michael Thy

**Affiliations:** Université Paris-Sud, Kremlin-Bicêtre, France; Infectious and Tropical Diseases Department, Bichat—Claude-Bernard Hospital, Assistance Publique—Hôpitaux de Paris, Université Paris Cité, Paris, France; Université Paris Cité and Université Sorbonne Paris Nord, INSERM, IAME, Paris, France; Infectious and Tropical Diseases Department, Bichat—Claude-Bernard Hospital, Assistance Publique—Hôpitaux de Paris, Université Paris Cité, Paris, France; Infectious and Tropical Diseases Department, Bichat—Claude-Bernard Hospital, Assistance Publique—Hôpitaux de Paris, Université Paris Cité, Paris, France; Infectious and Tropical Diseases Department, Bichat—Claude-Bernard Hospital, Assistance Publique—Hôpitaux de Paris, Université Paris Cité, Paris, France; Infectious and Tropical Diseases Department, Bichat—Claude-Bernard Hospital, Assistance Publique—Hôpitaux de Paris, Université Paris Cité, Paris, France; Bacteriology Laboratory, Hôpital Bichat—Claude-Bernard, Assistance Publique—Hôpitaux de Paris, Université Paris Cité, Paris, France; Cardiology Department, Hôpital Bichat—Claude-Bernard, Assistance Publique—Hôpitaux de Paris, Université Paris Cité, Paris, France; Infectious and Tropical Diseases Department, Bichat—Claude-Bernard Hospital, Assistance Publique—Hôpitaux de Paris, Université Paris Cité, Paris, France; Université Paris Cité and Université Sorbonne Paris Nord, INSERM, IAME, Paris, France; Infectious and Tropical Diseases Department, Bichat—Claude-Bernard Hospital, Assistance Publique—Hôpitaux de Paris, Université Paris Cité, Paris, France; Université Paris Cité and Université Sorbonne Paris Nord, INSERM, IAME, Paris, France; Infectious and Tropical Diseases Department, Bichat—Claude-Bernard Hospital, Assistance Publique—Hôpitaux de Paris, Université Paris Cité, Paris, France; Université Paris Cité and Université Sorbonne Paris Nord, INSERM, IAME, Paris, France; Infectious and Tropical Diseases Department, Bichat—Claude-Bernard Hospital, Assistance Publique—Hôpitaux de Paris, Université Paris Cité, Paris, France; Infectious and Tropical Diseases Department, Bichat—Claude-Bernard Hospital, Assistance Publique—Hôpitaux de Paris, Université Paris Cité, Paris, France; EA7323, Pharmacology and Drug Evaluation in Children and Pregnant Women, Université Paris Cité, Paris, France

## Abstract

**Introduction:**

*Enterococcus faecalis* is the third micro-organism causing endocarditis and is associated with a significant relapse rate. The objective of this study was to describe the management of patients with *Enterococcus faecalis* endocarditis (EE) and its implication for relapses.

**Methods:**

We conducted a monocentric, retrospective analysis of all patients hospitalized for EE including endocarditis or infection of cardiac implantable electronic device defined by the modified ESC 2015 Duke criteria in a referral centre in Paris, France.

**Results:**

Between October 2016, and September 2022, 54 patients with EE were included, mostly men (*n* = 40, 74%) with a median age of 75 [68–80] years. A high risk for infective endocarditis (IE) was found in 42 patients (78%), including 14 (26%) previous histories of IE, and 32 (59%) histories of valvular cardiac surgery. The aortic valve was the most frequently affected (*n* = 36, 67%). Combination therapy was mainly amoxicillin-ceftriaxone during all the curative antibiotic therapy duration (*n* = 31, 57%). Surgery was indicated for 40 patients (74%), but only 27 (50%) were operated on, mainly due to their frailty. Among the 17 deaths (32%), six (11%) happened during the first hospitalization for EE. A suppressive antibiotic treatment was initiated in 15 (29%) patients, mostly because of not performing surgery. During the 6-year study period an EE relapse occurred in three (6%) patients.

**Conclusions:**

EE is a worrying disease associated with a high risk of relapse and significant mortality. Suppressive antibiotic therapy could be a key treatment to limit the occurrence of relapses.

## Background

*Enterococcus* is the third cause of infective endocarditis (IE) worldwide, accounting for 5% to 20% of all cases, and the *Enterococcus faecalis* specie is responsible for 90% of *Enterococcal* endocarditis.^1–3^ The risk factors reported are an advanced age, diabetes, cancer, haemodialysis, cardiac implantable electronic device (CIED), prosthetic valve and the transcatheter aortic valve implantation (TAVI).^3–7^ The increase in the incidence of *Enterococcal faecalis* IE (EE) follows the modifications observed in the general epidemiology of IE, and notably the increased number of CIED, prosthetic valve and valve repair.^8,9^  *Enterococcus* is currently the second most common etiological agent of nosocomial endocarditis after *Staphylococcus*, with approximately 27% of hospital-acquired EE.^10^

An antibiotic combination with amoxicillin-gentamicin or amoxicillin-ceftriaxone is used to treat EE, the choice of the combination being up to clinician’s decision. Despite the absence of interventional study, the non-inferiority of amoxicillin-ceftriaxone has been demonstrated in multiple observational studies.^11–15^ Moreover, a key element in IE treatment is the importance of discussing a surgical intervention.^16^ Despite a significant risk of perioperative mortality, the long-term prognosis of patients after cardiac surgery seems improved, however, the surgery is often limited by the patient’s vulnerability factors (advanced age, comorbidities).^1,17,18^ For example, in the study by Habib *et al.*, among the 3116 IE included, cardiac surgery was indicated in 2160 (69%) patients, but finally performed in only 1596 (74% of them), leaving the remainder with potentially non-optimal treatment, and increased risk of rehospitalization or death.^19,20^ In a cohort of more than 500 IE, episodes with a clear indication for surgery but in which surgery was not performed were associated with significantly higher mortality (75.5% versus 20.2%, *P* < 0.001).^21^

Despite the improvements in treatment and diagnosis of IE (microbiological and nuclear-based imaging techniques),^19^ EE remains a serious disease associated with a mortality rate of 25% to 30% according to studies.^6,22^ As a comparison, mortality rate associated to *Streptococci* IE is of 16%,^1^ and to *Staphylococci* IE is of 44%.^3,6,7^ One of the main challenges in EE is the risk of relapse, which is particularly high for *Enterococcus* spp. compared to other causative agents of IE. EE has a relative risk of relapsing between 2 and 3 compared to *Staphylococcus* spp. and *Streptococcus* spp., with 7% of relapses for EE.^6,22^ In the recent study by Danneels *et al.*, surgery during treatment was a protective factor against 1-year relapse and death.^23^ To prevent relapse or death in the patients who cannot be operated despite having an indication for surgery, or who are at high risk of recurrence, suppressive antibiotic therapy is an interesting therapeutic option,^24,25^ but its efficacy remains to be demonstrated.

The aims of this study were to describe the systematic bundle management of patients with EE in a Parisian centre including surgery, combination therapy, search for the portal of entry and oral suppressive treatment and evaluate the rate of relapses.

## Methods

### Study design

We conducted a monocentric, retrospective cohort study from Bichat-Claude-Bernard University Hospital database (Paris, France). In this expert centre, all cases of IE are discussed with the Endocarditis Team. This multidisciplinary team is composed of cardiologists, cardiac surgeons, microbiologists, nuclear imaging specialists and infectious disease physicians, who rule together endocarditis management.

### Study population

We included all patients hospitalized in Bichat Hospital between 1 October 2016 and 1 September 2022 for possible or definite EE, including possible CIED-related IE, based on the ESC 2015 modified Duke criteria.^26^ The diagnosis of CIED-related IE was established when abnormal fixation was observed on imaging alongside meeting microbiological criteria for endocarditis, even in the absence of confirmed valvular infection on imaging, provided that at least three minor criteria for endocarditis were present (2015 ESC Guidelines).^16^ We excluded IE of other species than *Enterococcus faecalis* to focus on the outcome of this bacteria and because of the scarcity of other *Enterococcal* species. The follow-up began on the date of the first EE and ended either on the date of the end of follow-up (patient’s death or last visit to a hospital) or on 1 March 2023, for those still followed up at the end of the study. For patients lost to follow-up, the death status was verified through the INSEE registry.

### Bundle patient management

Antibiotic therapy was guided by 2015 ESC Guidelines.^16^ A combination therapy with amoxicillin associated either to ceftriaxone or gentamicin was used for 6 weeks, with sometimes switch to one another. The treatment could be prolonged in case of associated infection. No oral therapy was used for curative antibiotic therapy duration. Surgical indication was discussed for each patient with the Endocarditis Team. According to the 2015 ESC Guidelines, there are three main reasons to undergo surgery in the setting of acute IE: hemodynamic (heart failure including cardiogenic shock, pulmonary oedema or poor hemodynamic tolerance), uncontrolled infection [local complications (abscess, false aneurysm, fistula, enlarging vegetation), persistent positive blood cultures, resistant bacteria or fungi, prosthetic valve endocarditis (PVE) caused by *S. aureus* or non-HACEK Gram-negative bacilli] and prevention of septic embolization (in particular, to the CNS and for large vegetation > 10 mm). Reasons not to perform surgery, despite theoretical indication, could include patient frailty (old age, comorbidities increasing the risk of death during surgery), the difficulty of the surgery (defined as ‘risky surgery’ or dilapidated surgery) or the patient’s refusal. Portal of entry exploration was performed systematically for EE with cytobacteriological examination of urine and colonoscopy or positron emission tomography-computed tomography (PET-CT). Eradication of the portal of entry was performed each time it was possible. The indications for long-term suppressive oral therapy were discussed with the Endocarditis Team; one of these indications was a suboptimal curative treatment, but sometimes the suppressive antibiotic therapy was introduced for other reasons. This decision was taken through a multidisciplinary and individualized approach.

### Variables of interest

We collected the main characteristics of the patients, as well as data on the clinical presentation of the infection, complications and outcome, by reviewing all hospitalization records for each patient. We used the Charlson comorbidity index to categorize patients’ comorbidities. Data on relapse or death’s status were searched for each patient in the database software. A relapse was defined as a recurrence occurring up to 6 months after the end of antibiotic therapy, and a reinfection if the recurrence happened at 6 months or later.^27^

### Statistical analysis

Quantitative variables were expressed in medians and interquartile range (IQR) and categorical data in absolute numbers and proportions. The retrospective observational design of our study and the limited sample size did not enable us to conduct further statistical tests. Statistical analyses were performed using R software v.3.6.2.

### Ethics

An individual informed consent was not required for this anonymized register-based study. The study was approved by the Ethics Committee of the French Society of Infectious disease (IRB00011642, approval reference number 2023-0902). The study is in accordance with the General Data Protection Regulation (GDPR, EU law) and was recorded in the AP-HP (Assistance Publique-Hôpitaux de Paris) register.

## Results

### Main characteristics

Between 1 October 2016 and 1 September 2022, 54 patients with EE were included. The main characteristics of the patients are shown in Table 1. Overall, 32 patients had a history of surgical valvular replacement (59.3%).

**Table 1.** Baseline characteristics of the patients

Variables	*N* = 54—median [IQR]/number (%)
Age (years)	75.0 [67.7–79.9]
BMI (kg/m²)	26.0 [23.5–28.0]
Charlson score	2.0 [1.0–3.0]
***Past medical history***	
Endocarditis	14 (25.9)
*Enterococcal* endocarditis	7 (13.0)
Myocardial infarction	11 (20.4)
Congestive heart failure	5 (9.3)
Peripheric vascular disease	34 (63.0)
Stroke	6 (11.1)
Chronic pulmonary disease	6 (11.1)
Diabetes mellitus	14 (25.9)
Chronic kidney disease	9 (16.7)
Healed solid tumour	13 (24.1)
Evolutive solid tumour	2 (3.7)
Lymphoma	4 (7.4)
***Valvular disease***	38 (70.4)
**Aortic**	
Biological prosthetic valve	15 (27.8)
TAVI	7 (13.0)
Biological Bentall	1 (1.9)
Mechanical prosthetic valve	5 (9.3)
Mechanical Bentall	2 (3.7)
Aortic stenosis	1 (1.9)
Aortic insufficiency	2 (3.7)
**Mitral**	
Biological prosthetic valve	3 (5.6)
Mitral valvuloplasty	1 (1.9)
Mitral valvular disease	3 (5.6)
***No valvular disease***	16 (39.6)
***Habits***	
Tobacco (%)	14 (25.9)
Alcohol (%)	11 (20.4)
IV substance use (%)	2 (3.7)

BMI, body mass index; IV, intravenous.

### Initial presentation

According to 2015 modified Duke criteria, IE was definite in 40 cases and possible in 14 cases. The most common site of infection was the aortic valve (*n* = 36, 66.7%), mostly on prosthetic valves (*N* = 25, 69.4% of the aortic IE). Overall, there were 29 PVE, 53.7%), of which 7 (13.0%) were TAVI infections. The sites of infection are shown in Table 2.

**Table 2.** Site of infection

Site of infection	*N* (%)^a^
Aortic endocarditis	36 (66.7)
Prosthetic valve	18 (33.3)
Native valve	11 (20.4)
TAVI	7 (13.0)
Mitral endocarditis	18 (33.3)
Prosthetic valve	3 (5.6)
Native valve	15 (27.8)
Tricuspid endocarditis	2 (3.6)
Pulmonary endocarditis	1 (1.9)
Vascular prosthesis infection	4 (7.4)
Unknown	6 (11.1)

^a^Total greater than 100% because some patients had multiple sites of infection.

The Table S1 (available as Supplementary data at *JAC-AMR* Online) shows the results of diagnostic imaging. A transoesophageal echocardiography (TEE) permitted the diagnosis for five patients with no lesion on TTE (one abscess and four vegetations). Overall, a vegetation was found in 32 patients (59.3%), a valvular leak in 21 patients (38.9%), an aortic abscess in 11 cases (20.4%) and a stenotic valvular prosthesis in two patients (3.7%). The presence of an abscess on echocardiography was more frequent in PVE (eight patients, 27.6% of PVE) than in native valve endocarditis (NVE) (three patients, 12.0% of NVE). Among the 13 patients without lesion on TTE and TEE, nine (69.2%) had abnormal fixation on PET-CT, whereas leukocyte scintigraphy was normal in all these cases. Out of nine patients who underwent leucocyte scintigraphy three exhibited abnormal fixation. Subsequent PET-CT confirmed abnormal fixation in these three patients, and TTE revealed corresponding lesions. Secondary localizations were found in 31 patients (57.4%): cerebral in 12 cases (22.2%), splenic in eight cases (14.8%), osteoarticular in seven cases (13.0%) and pulmonary in five cases (9.3%) (Table S1).

### Management and evolution

**Figure 1.**
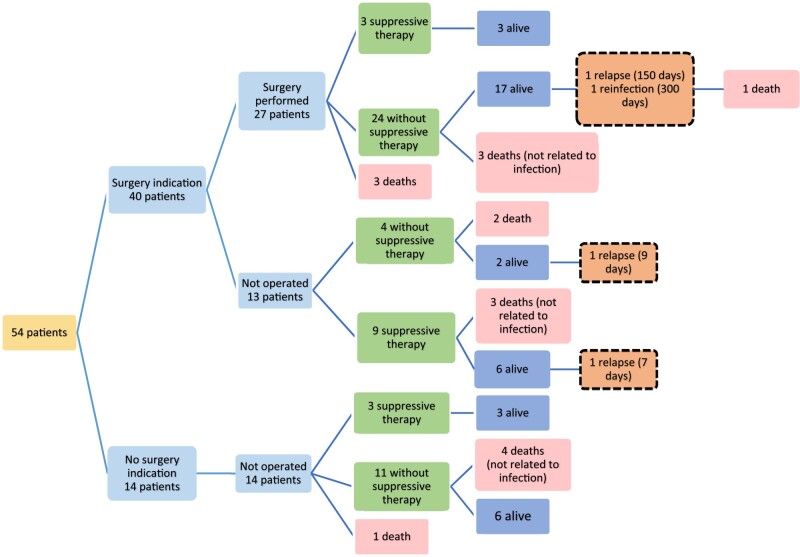
Management and outcomes.

The management of EE is shown in Table 3 and in Figure 1. Forty patients (74.1%) had a surgical indication, but only 27 (50.0% of the cohort) underwent surgery. The main reason for not performing surgery was the patient’s frailty. The main antibiotic combination was amoxicillin-ceftriaxone during all the curative antibiotic therapy duration (*n* = 31, 57.4%). The PET scan was performed at the end of curative treatment for 12 patients and motivated the implementation of suppressive antibiotic therapy for one patient, but it was not performed with the aim of stopping suppressive antibiotic therapy. The treatment duration was 42 days for 45 patients (83.3%), and the remaining nine patients had a longer curative antibiotic therapy, because of the non-favourable medical evolution, incomplete surgical treatment or the presence of osteo-articular associated infection. The portal of entry was searched for all patients but remained undocumented in 32 patients (59.3%). A digestive portal of entry was found in 10 patients (18.5%), urinary in six patients (11.1%) and dental in six patients (11.1%). Multidisciplinary discussion led to the prescription of suppressive antibiotic therapy in 15 patients (27.8%). Six of the nine patients with longer curative antibiotic therapy had a prescription of suppressive antibiotic therapy (66.7%). The prescription was primarily motivated by the absence of surgery despite a theoretical indication in most cases (eight patients, 14.8%). In other instances, the decision was influenced by a high risk of relapse due to factors such as incomplete surgery (one patient), persistence of the portal of entry (one patient), a previous episode of *Enterococcal* endocarditis (one patient) and other comorbidities posing a high risk of relapse (TAVI) (three patients). No adverse effect or observance issue have been reported during the follow-up, and there was no treatment discontinuation. Patients on suppressive therapy seemed older than patients who were not [median ages 79.0 (66.5–86.2) years old versus 75.0 (68.7–78.2) years old].

**Table 3.** Management of Enterococcal endocarditis

Variables	*N* (%)
Curative antibiotic therapy	
Amoxicillin-ceftriaxone	31 (57.4)
Amoxicillin-gentamicin (1–2 weeks) then amoxicillin-ceftriaxone	5 (9.3)
Amoxicillin-gentamicin (2 weeks) then amoxicillin-ceftriaxone	4 (7.4)
Other	14 (25.9)
Surgical indication	40 (74.1)
Infectious	17 (31.5)
Embolic	20 (37.0)
Hemodynamic	7 (13.0)
Surgical procedure	27 (50.0)
Aortic valve replacement	12 (22.2)
Aortic and mitral valve replacement	7 (13.0)
Mitral valve replacement	3 (5.6)
Bentall implantation	2 (3.7)
Other	1 (1.9)
Missing	2 (3.7)
Reasons of no surgery despite surgical indication	13 (24.1)
Comorbidities	8 (14.8)
Risky surgery	4 (7.4)
Patient’s refusal	1 (1.9)
42 days of curative antibiotic therapy	45 (83.3)
Suppressive antibiotic therapy	15 (27.8)
Amoxicillin 2000 mg/day	14 (25.9)
Amoxicillin 3000 mg/day	1 (1.9)
Length of hospital stay (days): median [IQR]	36.5 [25.0–49.8]
Length of follow-up (months): median [IQR]	14.4 [3.1–37.8]

### Recurrent endocarditis

Seven patients had a previous history of EE before the inclusion, without prescription of suppressive antibiotic therapy at that time. For five of them, the episode of IE considered for inclusion in this study was already a relapse of a previous episode due to the same micro-organism that occurred within 6 months before the inclusion. All had a prosthetic aortic valve or a TAVI and four of them had in addition a CIED. During the current episode, all had an indication for surgery, but only three underwent surgery.

### Outcomes

After a median time of follow-up of 14.4 months (3.1–37.8), there were four recurrences including three relapses (5.6%) and one reinfection, all with *Enterococcus faecalis*. The characteristics of these patients are shown in Table 4. One of these patients was under suppressive therapy at the time of the relapse, but only for 7 days (the relapse occurred seven days after the end of curative antibiotic therapy). Two of the three relapses occurred in patients with previous history of EE (so the relapse was the third episode of EE).

**Table 4.** Description of patients with recurrence

Age/gender	Comorbidities	Endocarditis	Treatment	Delay	Number of relapses
83.6/M	5 previous endocarditis PM and biologic aortic valve Stroke, HTA, healed solid tumour	Aortic and mitral	Aortic and mitral replacement Amoxicillin—ceftriaxone No suppressive antibiotic therapy	157	2
83.4/M	0 previous endocarditis Leak of mitral valve HTA, chronic pulmonary disease, hepatopathy, diabetes	pulmonary	Indication but no operated Amoxicillin- ceftriaxone No suppressive antibiotic therapy	9	2
76.4/F	0 previous endocarditis HTA, kidney transplantation	aortic	Biologic aortic replacement Amoxicillin-ceftriaxone No suppressive antibiotic therapy	300	1
79.0/M	1 previous endocarditis Biologic aortic valve HTA, diabetes	aortic	Indicated but no operated Amoxicillin-gentamicin and amoxicillin- ceftriaxone Suppressive antibiotic therapy	7	1

PM, Pacemaker.

At the time of hospital discharge, 19 patients (35.2%) directly went home, and the others went through rehabilitation. Six patients (11.1%) died during the first hospitalization for IE. By the end of follow-up, 17 patients (31.5%) were dead (Figure 1). Seven deaths were directly due to EE (13.0% of the cohort).

## Discussion

In this study including 54 EE, 17 patients died (31.5%) and three patients had a relapse (5.6%). Two of these three patients had a theorical indication of surgery for the first EE episode but could not be operated. In our cohort, there was a notably high proportion of PVE, reflecting the epidemiology of this disease affecting an ageing and comorbid population. This proportion was higher in comparison to other studies,^2,3^ due to Bichat Hospital being a referral centre for cardiac surgery. The occurrence of abscesses detected through echocardiography at the time of diagnosis was more frequent in PVE cases when compared to NVE, consistent with the observations made by Anderson *et al.* Their study noted that 20% of PVE cases and 6% of NVE cases exhibited abscesses.^2^ This disparity can be explained by the intrinsic difficulty in sterilizing PVE, often necessitating surgical intervention. Moreover, our cohort displayed a prevalence of aortic valve involvement in 66.7% of cases, in line with the findings of McDonald *et al.*^7^

Our sample was representative of the population of patients affected with EE, with elderly and comorbid patients, and a high risk of relapse.^28^ In a cohort of 279 EE, the rate of relapse was even higher (9.3%), maybe because of a different definition (all recurrences within 1 year of the first infection were considered relapses in this study).^23^ We chose a more restrictive definition because we could not control the similarity of the micro-organism by genotypic analyses and to avoid overestimating relapses. The number of deaths was notably elevated (31.5%) at the end of follow-up, with regards to findings from other studies where rates were 26.5%^23^ and 23.0%.^1^ This high mortality rate could be partially attributed to the transfer of severely ill patients from other centres who required surgical intervention, Bichat Hospital being a specialized centre with surgical proficiency.

Seven patients in the cohort had a history of EE; consequently, five EE included were actually relapses. We excluded them from analysis in our study because we did not have enough information about these previous infections, especially their initial management, and because we did not have access to this past medical history for every patient. Although the number of relapses is probably underestimated here, these data underline the importance of relapses after EE, some patients having not only one relapse but several. This high rate of relapses with *Enterococcus faecalis* endocarditis may have multiple explanations; first, EE are associated with older and more comorbid patients than non-EE, limiting the possibility of performing surgery when indicated^6^ and surgery is a protective factor from relapse.^29^ Second, the growing rate of recurrences falls within the context of increasing treatment with amoxicillin and ceftriaxone that has a reduced renal toxicity compared to amoxicillin plus gentamicin. However, the amoxicillin and ceftriaxone combination does not seem to be associated with higher risk of relapse.^29^ Finally, in our study, the portal of entry was not identified in more than 50% of cases, and so could not be treated, maintaining a potential source of *Enterococcus faecalis* bacteraemia.

In our cohort of 54 patients, 15 had been prescribed suppressive antibiotic therapy, with good compliance and tolerance. One of them had a relapse, but since it occurred 7 days after discontinuation of curative therapy, it could be considered as a failure of initial curative treatment rather than a relapse. To our knowledge, this is the largest cohort of EE patients on suppressive antibiotic therapy. A retrospective study in Spain included 32 IE with surgical indication but no operation performed, on long-term (>8 weeks) antibiotic therapy. Among them, 23 patients had a prescription of life-long oral antibiotic therapy (4 EE), that was beta-lactams, trimethoprim-sulfamethoxazole or other agents. Four of these patients experienced a relapse during the 8 years follow-up, but one of them had stopped the suppressive therapy, and another one had developed a resistance.^30^ At the time of writing, there is no recommendation for suppressive therapy and, through our work, it seems this treatment could be a key treatment to avoid relapses, especially in patients with a higher risk of recurrence.

Our study has several limitations. First, it is a retrospective cohort based on database records, leading to missing values. Since detailed information about history of endocarditis was often lacking, the rate and the distinction between relapse and reinfection were difficult to assess. The number of relapses could have been underestimated. Moreover, because the date of analysis was close to the date of the end of inclusions, we probably have missing values concerning the portal of entry, sometimes searched months after the initial infection. Then, it is a monocentric study with a small sample size, permitting only descriptive statistics. Finally, the Bichat Hospital is an expert centre for the management of IE and include particularly severe cases; our results should not be generalized to all EE.

### Conclusion

*Enterococcal* endocarditis is a growing disease affecting elderly and comorbid patients, often limiting surgery when indicated and leading to a high rate of relapse. To face this problem, the systematic investigation of the portal of entry is essential and the suppressive antibiotic therapy could help, but it should be evaluated in larger and prospective studies.

## Supplementary Material

dlae033_Supplementary_Data

## Supplementary data

Table S1 is available as Supplementary data at *JAC-AMR* Online.
