# Supplementary material for: Enterococcal endocarditis management and relapses
Source: JAC Antimicrob Resist. 2024 Mar 6;6(2):dlae033. doi: 10.1093/jacamr/dlae033 (PMC10915900; doi:10.1093/jacamr/dlae033)
Supplement: dlae033_Supplementary_Data [file dlae033_supplementary_data.docx]

# Supplementary Data

**Table S1: Diagnostic imaging**

| **Imaging** | **Number (%) *** |
| --- | --- |
| **TTE** | 52 (96.3) |
| Endocarditis lesion | 31 (57.4) |
| Vegetation | 22 (40.7) |
| Leak | 16 (29.6) |
| Abscess | 8 (14.8) |
| Stenosing prosthesis | 2 (3.7) |
| No lesion | 14 (25.9) |
| Missing | 7 (13.0) |
| **TEE** | 41 (75.9) |
| Endocarditis lesion | 34 (63.0) |
| Vegetation | 26 (48.1) |
| Leak | 16 (29.6) |
| Abscess | 10 (18.5) |
| Stenosing prosthesis | 1 (1.9) |
| No lesion | 6 (11.1) |
| Missing | 1 (1.9) |
| **PET-CT** | 31 (57.4) |
| Fixation | 22 (40.7) |
| PVE | 15 (27.8) |
| NVE | 7 (13.0) |
| **Leucocyte scintigraphy** | 9 (16.7) |
| Fixation | 3 (5.6) |
| **Cardiac CT** | 6 (11.1) |
| **Vegetation size (mm) - Median [IQR]** | 13.0 [10.0-15.3] |
| **Embolic events or metastatic infection** | 31 (57.4) |
| Cerebral | 12 (22.2) |
| Splenic | 8 (14.8) |
| Bones and articulations | 7 (13.0) |
| Pulmonary | 5 (9.3) |
| Other | 8 (14.8) |

*Total greater than 100% because some patients had multiple lesions
